# Audiovisual Multisensory Integration and Evoked Potentials in Young Adults With and Without Attention-Deficit/Hyperactivity Disorder

**DOI:** 10.3389/fnhum.2019.00095

**Published:** 2019-03-19

**Authors:** Heather S. McCracken, Bernadette A. Murphy, Cheryl M. Glazebrook, James J. Burkitt, Antonia M. Karellas, Paul C. Yielder

**Affiliations:** ^1^Faculty of Health Sciences, University of Ontario Institute of Technology, Oshawa, ON, Canada; ^2^Faculty of Kinesiology and Recreation Management, University of Manitoba, Winnipeg, MB, Canada; ^3^Health, Leisure & Human Performance Institute, University of Manitoba, Winnipeg, MB, Canada; ^4^Faculty of Health, School of Medicine, Deakin University, Waurn Ponds, VIC, Australia

**Keywords:** ADHD, multisensory integration, EEG, event-related potentials, response time

## Abstract

The purpose of this study was to assess how young adults with attention-deficit/hyperactivity disorder (ADHD) process audiovisual (AV) multisensory stimuli using behavioral and neurological measures. Adults with a clinical diagnosis of ADHD (*n* = 10) and neurotypical controls (*n* = 11) completed a simple response time task, consisting of auditory, visual, and AV multisensory conditions. Continuous 64-electrode electroencephalography (EEG) was collected to assess neurological responses to each condition. The AV multisensory condition resulted in the shortest response times for both populations. Analysis using the race model (Miller, 1982) demonstrated that those with ADHD had violation of the race model earlier in the response, which may be a marker for impulsivity. EEG analysis revealed that both groups had early multisensory integration (MSI) occur following multisensory stimulus onset. There were also significant group differences in event-related potentials (ERPs) in frontal, parietal, and occipital brain regions, which are regions reported to be altered in those with ADHD. This study presents results examining multisensory processing in the population of adults with ADHD, and can be used as a foundation for future ADHD research using developmental research designs as well as the development of novel technological supports.

## Introduction

Attention-deficit/hyperactivity disorder (ADHD) is a common neurodevelopmental disorder. The most common symptoms associated with a diagnosis of ADHD are hyperactivity, impulsivity, and inattention (Visser et al., 2014). These symptoms typically arise during childhood, with approximately 11% of children receiving a diagnosis of ADHD (Visser et al., 2014). Although ADHD is often associated with being a predominately childhood disorder, it is commonly present in the adult population (Wilens et al., 2004). Specifically, of the children diagnosed with ADHD, approximately 50% will have symptoms persist into adulthood (Sadock et al., 2000). Adult males are more commonly diagnosed with ADHD than adult females (5.5% vs. 2%; Amiri et al., 2014). Although the diagnosis of ADHD is based on behavioral characteristics, neurological characteristics have been reported.

Those with ADHD have been found to have altered brain structures through the utilization of functional magnetic resonance imaging (fMRI) and electroencephalography (EEG; Bresnahan and Barry, 2002; Castellanos et al., 2002; Makris et al., 2007; Proal et al., 2011; Duerden et al., 2012). For instance, general cerebral gray matter reductions are present in adults (Makris et al., 2007), along with a diffuse pattern of thinning in parietal, temporal, frontal, occipital, and cerebellar cortices in children and adults with ADHD (Castellanos et al., 2002; Valera et al., 2007; Proal et al., 2011; Duerden et al., 2012). Thicker gray matter is reported in the pre-supplemental motor area and in the right hemispheric primary somatosensory cortex (Duerden et al., 2012). The presence of alterations to these brain structures indicates that there may be associated alterations to the functions related to these regions as well. In particular, multisensory integration (MSI) has been shown to involve the parietal and occipital cortical regions, and these regions are altered in those with a diagnosis of ADHD (Makris et al., 2007; Brandwein et al., 2011; Proal et al., 2011).

MSI is the process by which the sensory systems work together to form a unified perception of the external world (Stein and Wallace, 1996). This sensory process is crucial to how one interacts with and perceives their environment. In order to make sense of various afferent inputs it is necessary for the nervous system to effectively process these incoming stimuli. MSI can result in optimized behavioral performance enhancements, such as shorter response times and greater response accuracy (Meredith et al., 1987; Laurienti et al., 2004). For sensory cues to be processed as multisensory and result in neural and behavioral enhancements, it is important for the components to be semantically congruent and to occur simultaneously or with a slight timing offset (Driver and Spence, 2000; Laurienti et al., 2004). If sensory conditions are not, this can result in sub-optimal performance, resulting in greater response latencies (Laurienti et al., 2004).

Audiovisual (AV) MSI involves the specific integration of auditory and visual stimuli that occur close in temporal and spatial proximity. This form of sensory integration occurs throughout daily life. When in a classroom setting the nervous system is constantly processing all of the auditory stimuli from things that one is hearing as well as all of the visual stimuli from things that they are seeing. Since these stimuli most often occur in close temporal and spatial proximity, AV MSI contributes to the formation of coherent perceptions, such as when detecting and identifying a presented stimulus (Foxe and Molholm, 2009). Previous studies have suggested that alterations to AV MSI are associated with impairments in communication and sensory processing when in social settings (Brandwein et al., 2013, 2015).

A simple response time task has been effectively used to assess MSI (Brandwein et al., 2011, 2015). This task consists of providing participants with multiple stimulus conditions (e.g., auditory unisensory, visual unisensory, and AV multisensory) that all represent the color red. When a participant is presented with any of the stimulus conditions, the same response is required (e.g., click of a button with the right thumb). This is a simple response time task because the same response is required for each stimulus condition, meaning participants do not have to dissociate a certain response with a specific stimulus (as is seen in a two-alternative forced-choice discrimination task). Demonstrating shorter simple response latencies in the multisensory vs. unisensory conditions allows for a behavioral assessment of MSI. Response times can also be examined with Miller’s race model, which examines the probability that faster response times in multisensory conditions arise because of MSI, as opposed to the faster of the two unisensory stimuli triggering the response. Accordingly, race model violation means that MSI likely occurred (Miller, 1982). While this simple response time task provides a behavioral examination of MSI, EEG can be used to examine its neurophysiology (Stevenson et al., 2014).

EEG is used to measure cortical activity, and can be employed to assess the neurophysiology of MSI through a method known as the principle of superposition of electrical fields. This principle states that any significant divergence between a multisensory waveform and a “sum” waveform, which is derived from summing the auditory and visual unisensory EEG waveforms, represents that MSI is occurring (Molholm et al., 2002; Brandwein et al., 2011). When the sum and multisensory waveforms differ significantly, it suggests the two sensory inputs are being integrated and processed differently than when they are presented individually, resulting in different EEG waveforms (Stevenson et al., 2014). Previous studies utilizing this EEG methodology have noted that there are specific brain regions involved in MSI, most notably the parietal region (Molholm et al., 2006; Moran et al., 2008; Brandwein et al., 2011, 2015), a region known to be structurally altered in those with ADHD (Proal et al., 2011). However, although these brain alterations suggest that MSI may be altered in those with ADHD, no research has been conducted to assess AV MSI in this population.

Considering that ADHD is commonly described as a childhood disorder and despite its prevalence in adulthood, literature pertaining to adult ADHD is lacking. However, no research has yet investigated whether AV MSI is altered in any way in those who have received a diagnosis of ADHD. Therefore, the purpose of the present study was to examine whether young adults who have received a clinical diagnosis of ADHD at some point in their lives have altered MSI compared to neurotypical controls. The findings will help to elucidate if and when MSI occurs in both groups through simple response time differences and divergence of EEG waveforms (i.e., sum vs. multisensory) in AV multisensory vs. unisensory conditions. We hypothesized that due to the altered brain structure in regions involved in multisensory processing that MSI would occur differently in those with ADHD compared to controls; particularly in regions known to have altered structure in those with ADHD, i.e., the parietal and occipital regions. Further, we hypothesized that these neurological differences would be reflected in behavioral differences in MSI.

## Materials and Methods

### Participants

This study was carried out in accordance with the recommendations of the University of Ontario Institute of Technology (UOIT) Research Ethics Board (REB) and participants gave written informed consent prior to participating. This study was performed according to the principles set out by the Declaration of Helsinki for the use of humans in experimental research. Participants were recruited from the student body at the UOIT. Recruitment was done through the use of word of mouth, in-course announcements, and posters placed throughout the campus. Participants recruited were young adults between the ages of 18–35 years old that had and had not received a clinical diagnosis of ADHD at some point in their life. Adults that reported receiving a diagnosis of ADHD, self-reported the age at which they were diagnosed as well as any medication that was currently being taken to control their symptoms related to ADHD. The mean age for neurotypical controls (*n* = 11, three females) was 21.3 ± 3.0 years old and for the ADHD group (*n* = 10, three females) was 24.1 ± 3.5 years old. The mean age of ADHD diagnosis was 13.1 ± 7.4 years old.

The Edinburgh Handedness Questionnaire was used to determine which hand was the most dominant per participant, with the results indicating left, right, or ambidextrous. This was completed because the stimulus response was done with the right thumb. The number of left-handed participants per group was similar; so that any potential differences in behavioral, electrophysiological, or movement time were not related to a handedness-bias since the right hand was not the dominant limb for each participant. The neurotypical control group had three left-handed, seven right-handed, and one ambidextrous participant while the ADHD group had two left-handed, three right-handed, and five ambidextrous participants.

The adult ADHD Self-Report Scale (ASRS-v1.1) was used to assess each participant’s symptoms associated with ADHD. The ASRS has a total of 18 questions, which are in line with the ADHD diagnostic criteria set out in the DSM-IV (Dankner et al., 2017), and is rated on a 5-point Likert scale ranging from “never” to “very often” for each question. This screening tool is highly sensitive for predicting ADHD symptomatology (van de Glind et al., 2013). This was included to ensure that we did not include participants with potential ADHD in the control group, and equally that we did not include a participant in the ADHD group whose symptoms may have resolved. Those with ADHD almost always selected “sometimes,” “often,” or “very often” with respect to individual questions, whereas the neurotypical control group selected “never” or “rarely” for almost each question. Participants were also asked to report whether they were currently taking medication for their ADHD. Six participants with ADHD reported that they were taking medication for ADHD at the time of participation. Medications reported included Vyvanse, Concerta, and Adderall. Participants were instructed to maintain their normal medication dosage and timing, as to mimic their functional capabilities on a typical day.

Participants completed pre-screening questionnaires prior to beginning the research protocol. An EEG safety checklist was completed to ensure that participants did not have any experiences or conditions that may be contraindicated for the collection of EEGs. This includes a recent history of epilepsy, concussion, stroke, or brain injury, which may potentially alter the results and make the task unsafe for participation.

### Stimuli

#### Auditory-Alone

An audible female voice was presented speaking the word red (duration ~180 ms) from speakers placed bilaterally to the computer screen. The volume control was adjusted to the halfway mark to ensure a comfortable and easily discernible sound level, which was the same volume for each participant.

#### Visual-Alone

A red circle (diameter 30 cm) appeared on the screen for 60 ms, placed centrally in the vertical and horizontal plane.

#### Multisensory

The auditory and visual stimuli occurred simultaneously from speakers and a computer screen adjacent to one another.

### Procedures

**Figure 1:**
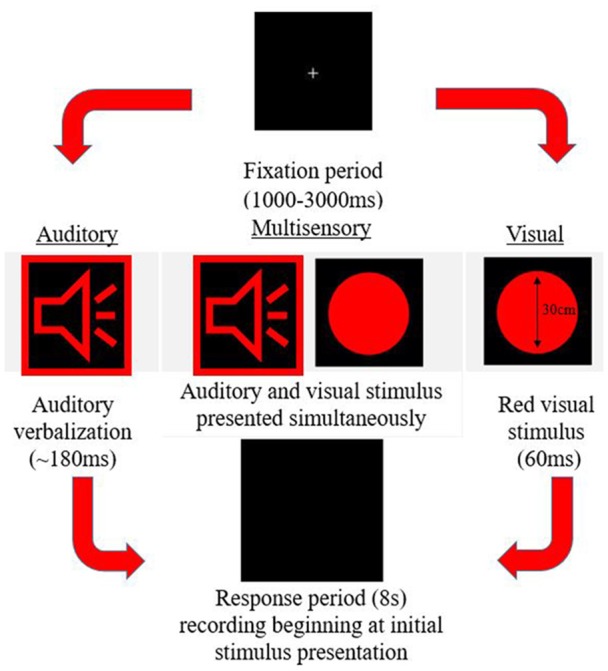
Example of the three possible stimulus conditions within the simple response time task.

A simple response time task was utilized to measure MSI. This paradigm was designed using E-Prime 2.0 Professional (Psychology Software Tools, Inc., Sharpsburg, PA, USA). The task consisted of three different stimuli conditions (visual, auditory, and multisensory) all presented in random order with an inter-trial interval of 1,000–3,000 ms. The trial events are depicted in Figure 1. Stimuli were presented in eight blocks, with each block consisting of 102 stimuli (34 per condition). The same response was required for each condition previously described, ensuring that there were no complex decision-making processes necessary for a response, which would otherwise slow the response. Participants were instructed to respond with their right thumb. A Chronos^®^ (Psychology Software Tools, In., Sharpsburg, PA, USA) response device was used to receive and collect responses. Response time was calculated as the time from stimulus onset to when a button press response on the device was made. This device was used for its 1 ms temporal accuracy.

### Data Acquisition and Analysis

#### Behavioral

E-Prime 2.0 Professional (Psychology Software Tools, Inc., Sharpsburg, PA, USA) was utilized to run, collect, and record responses. While performing the simple response time task on a desktop computer, continuous EEG was recorded.

#### ERPs

A Waveguard™ 64-electrode EEG cap (ANT Neuro, Netherlands) was used to collect surface brain electrical activity in response to each stimulus type. The use of a 64-electrode cap allows for a more robust analysis of brain activity, as acquisition is not limited to a few electrodes. The Waveguard™ cap was connected to a TMSi REFA-8 amplifier with 64 EEG channels, four bipolar channels, and four auxillary channels; which was run through asaLab™ (Netherlands) to collect and record each session at a 2,048 Hz sampling frequency. Event-related potential (ERP) analysis was completed on a separate laptop using Advanced Source Analysis (ASA™; Netherlands), Matlab™ (Natick, MA, USA), and SPSS^®^ (Armonk, New York, NY, USA).

Data was cleaned and removed of any artifacts prior to running any analyses. Artifacts which were a result of muscle activity and ocular activity were removed. EEG data was filtered using a band-pass filter with a low cut-off of 1.6 Hz and a high cut-off of 45 Hz and a slope of 24 db/octave was utilized. The low cut-off of 1.6 removes any slow-wave activity that would otherwise be represented twice in the “sum” waveform during analysis. The 45 Hz high cut-off removes any artifacts that are a result of surrounding electrical equipment. Artifact rejection was performed, with the exclusion criteria being ±100 μV. Finally, data for each trial were averaged into 600 ms epochs per participant per condition, which included 100 ms pre-stimulus and 500 ms post-stimulus-onset (total duration of 600 ms). Average waveforms for each unisensory condition were summed (auditory + visual) for comparison to the multisensory waveform (Foxe et al., 2000; Molholm et al., 2002; Brandwein et al., 2011, 2013, 2015). This was done in accordance with the principle of superposition of electrical fields and nonlinear summation. Based on this principle, any significant divergence between the sum and multisensory waveform indicates that the two stimuli presented simultaneously interacted and were processed differently than their unisensory condition counterparts. When completing the analysis, any significant divergence between these two waveforms would indicate if and when MSI occurred, and whether the pattern of MSI was different between the two cohorts (ADHD vs. neurotypical). In areas and time points where divergence between the two waveforms was significant (greatest difference from 0 μV) it can be inferred that MSI was occurring. Consistent with previous studies, latencies and electrodes for MSI ERP analysis were chosen based upon the grand-averaged head models where the greatest positive and negative activity occurred at various latencies (Russo et al., 2010; Brandwein et al., 2011, 2013), which can be seen in the included figures. A strength of this method is that it finds the regions of maximal positivity and negativity in a given time window. These same waveforms could be picked up by a number of other electrodes on the scalp at similar latencies. By selecting the group of electrodes with the maximal positivity and negativity for a given time window, it sets objective criteria for analyses, and ensures that we are not re-analyzing the same waveform differences at multiple electrode sites. An analogy is the 12-lead electrocardiogram (ECG) where the same cardiac activity can be seen between multiple different electrode pairs but there are certain pairs that are best for imaging different regions of the heart’s electrical conducting system.

### Statistical Analyses

#### Behavioral

Mean response times were calculated per participant in response to each stimulus type (auditory alone, visual alone, and AV multisensory). Outliers were removed, and any responses that were within ±2 SDs from their individual condition average were included when calculating each participant’s average per condition, with the caveat being that the lower limit could not be any faster than 100 ms; for participants where –2 SDs was in fact lower than 100 ms, the lower limit was then set to 100 ms. A 2 group (ADHD, neurotypical) by 3 sensory condition (A, V, multisensory) mixed factors analysis of variance (ANOVA), with repeated measures on the last factor, was used to determine whether there were any significant differences in response time dependent on diagnostic status and/or sensory condition. Alpha was set as *p* < 0.05. Partial eta-squared (*η*^2^) was used to report effect size, where a small effect was noted as 0.01, medium as 0.06, and a large effect as 0.14 (Richardson, 2011). Tukey’s Honestly Significant Difference *post hoc* tests were used to decompose significant mean differences involving more than two means. All statistical tests were run using SPSS^®^ (Armonk, New York, NY, USA) version 24 (Nie et al., 1970). All tests were checked for normality using Shapiro-Wilk’s test and homogeneity of variance using Levene’s test.

#### Race Model

Miller’s “race model” was applied (Miller, 1982; Ulrich et al., 2007) to test whether any potential improvements, or gain, in response times in the multisensory condition reflected MSI. This was completed using the MATLAB™ (Natick, MA, USA) algorithm provided by Ulrich et al. (2007).

Miller’s race model places an upper limit cumulative probability on a reaction time occurring in response to redundant stimulus pairs (i.e., a visual and auditory multisensory stimulus representative of “red”) within a given latency window, or quantile. This test is based upon statistical probability and cumulative density functions (CDFs) of responses acquired from the redundant multisensory condition as compared to the two unisensory conditions. The purpose is to determine whether improvements in response time that occur in the multisensory conditions results from MSI, as opposed to the faster of the two unisensory stimuli triggering the response (Ulrich et al., 2007; Brandwein et al., 2015). For multisensory integrational purposes, the race model is considered violated when the multisensory percentile score is significantly less than the predicted race model percentile score within a given probability quantile (Ulrich et al., 2007). This is expected to occur at the probability quantiles associated with earlier response times (i.e., when the multisensory curve is to the left of the race model curve with time on the *x*-axis and quantiles on the *y*-axis. This is representative of sensory integration at earlier response latencies as this is when the integration of unisensory inputs is expected to fulfill the multisensory response criterion, before either unisensory input is independently processed (Miller, 1982; Ulrich et al., 2007). In other words, the response to the multisensory condition is faster than what the race model predicts. This results in behavioral gains through faster responses and is known as the redundant signals effect (RSE; Ulrich et al., 2007).

All calculations and tests of the race model were performed using the MATLAB™ (Natick, MA, USA) script provided by Ulrich et al. (2007). It should be noted that failure to violate the race model does not necessarily mean that sensory conditions did not interact when presented synchronously; it simply means that the response facilitation that occurred can be described and predicted based on probability summation of the two unisensory conditions (Brandwein et al., 2011). Race model violation was assessed for both the ADHD and neurotypical control groups, using paired samples *t*-tests to compare the multisensory and race model scores at probability quantiles ranging from 0.05% to 0.95% in 10% increments. Significant (*p* ≤ 0.05) deviations for each response quantile were identified (Laurienti et al., 2006; Farid et al., 2018).

#### ERPs

All ERP processing was done offline using ASA (Netherlands), Matlab™ (Natick, MA, USA), and SPSS^®^ (Armonk, New York, NY, USA) software. As has been done in the previous literature (Giard and Peronnet, 1999; Molholm et al., 2002; Brandwein et al., 2011, 2013, 2015), multisensory interactions were analyzed by comparing the AV multisensory waveform to a “sum” waveform [AV − (A + V)]. The sum waveform was created by summing the two unisensory conditions (A and V). Based on the principle of superposition of electrical fields, any significant divergence between the MSI and sum waveform (AV ≠ A + V) suggests that MSI did occur, or in other words, that the two simultaneously occurring stimuli interacted (i.e., were not processed in a unisensory fashion). In order to not bias the analysis of the dependent measures (difference between multisensory and sum waveform per group) to areas where there may be EEG differences between those with and without ADHD, the electrodes and time frames for analysis were chosen based on an overall grand-average heat map for the AV multisensory stimulus. This grand average was created using all participants’ data (ADHD and neurotypical) as not to bias regions of interest to one group’s activity. Time windows for analysis were constrained between 0 and 250 ms following stimulus onset, which reflect early multisensory interactions (Brandwein et al., 2011). For each time frame and region chosen for analysis, averaged-data per participant was added to a 2 group (ADHD, neurotypical) by 2 stimulus type (MSI, sum) mixed factors ANOVA with repeated measures on the last factor, were used to examine multisensory processing in the ADHD and neurotypical control groups. Scalp regions were represented by an average of 2–4 composite electrodes, being the electrodes that showed greatest activity during that time frame. The regions and time-frames chosen are similar to those discussed in previous literature and are presented in Table 1 (Giard and Peronnet, 1999; Foxe et al., 2000; Molholm et al., 2002; Brandwein et al., 2011, 2013, 2015). Alpha for all analyses was set to *p* < 0.05 and Greenhouse-Geisser corrections were used when appropriate to correct for violations of sphericity. All statistical tests were completed using SPSS^®^ (Armonk, New York, NY, USA) version 24 (Nie et al., 1970). All tests were checked for normality via Shapiro-Wilk’s test and homogeneity of variance using Levene’s test.

**Table 1:** Time windows and electrodes used for analyses.

Region (electrodes)	Time windows
Central parietal (CPz and Pz)	100–140 ms
Parietal occipital (PO7, O1, O2, and PO8)	100–120 ms
Prefrontal (FPz, FP2, and FP1)	110–120 ms
Parietal occipital (Pz, P1, and POz)	140–160 ms

## Results

### Behavioral

**Figure 2:**
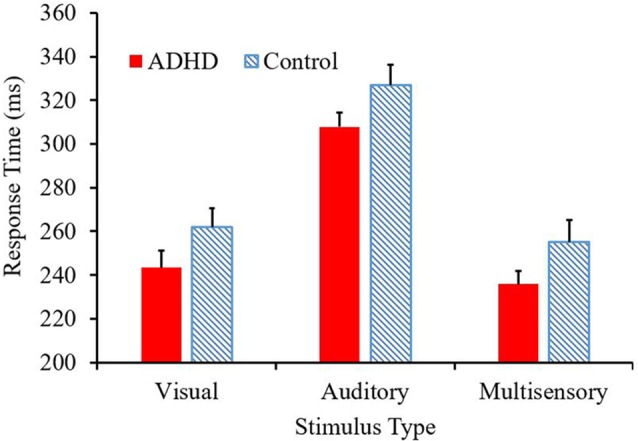
Average response time (ms) per condition, with attention-deficit/hyperactivity disorder (ADHD) responses represented in red and controls in the blue pattern.

The mean response time for both the ADHD and control group are presented in Figure 2. A main effect of stimulus condition *F*_(2,38)_ = 587.89, *p* < 0.001, partial *η*^2^ = 0.969, revealed that responses to the multisensory stimulus were significantly faster than responses to either of the unisensory conditions (see Figure 2). Although there is a trend toward those with ADHD to respond faster to each stimulus type (308 ms vs. 327 ms; 243 ms vs. 262 ms; 236 ms vs. 255 ms) compared to their neurotypical counterparts, a significant group effect was not reached (*F*_(1,19)_ = 2.709; *p* = 0.116; partial *η*^2^ = 0.125). There was no group by stimulus condition interaction (*F* = 0.021).

### Race Model

**Figure 3:**
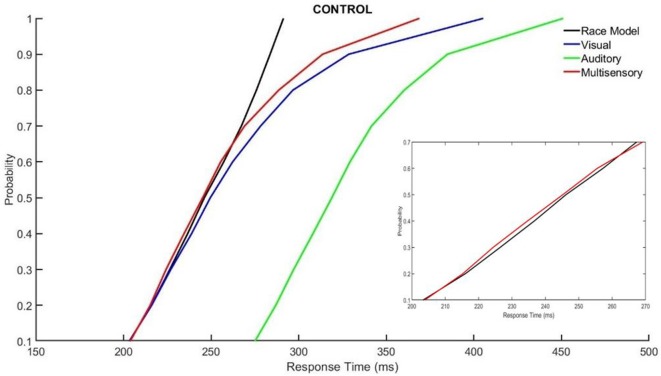
Cumulative probability quantile comparisons between the multisensory and race model scores for neurotypical controls.

**Figure 4:**
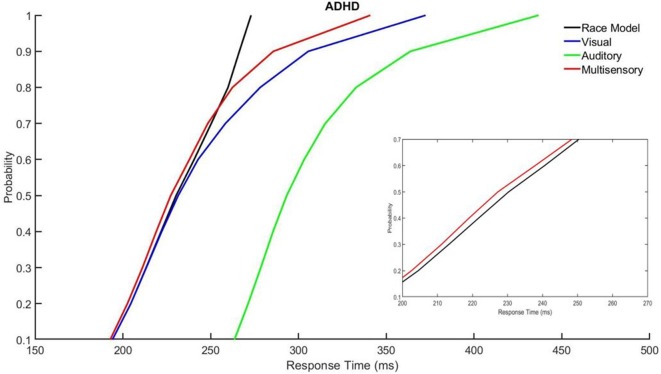
Cumulative probability quantile comparisons between the multisensory and race model scores for participants with ADHD.

Those with ADHD showed violation of the race model in the 5th quantile, *t*_(9)_ = 2.81, *p* = 0.020, while controls did not (Figures 3, 4).

### Neurophysiological

When assessing the AV multisensory responses, various distinct patterns of centralized activity were found in specific locations and at specific latencies. Latencies assessed were between 0–250 ms. These time windows and areas of greatest activity were used to assess whether MSI was occurring in both study groups based on the principle of superposition of electrical fields.

**Figure 5:**
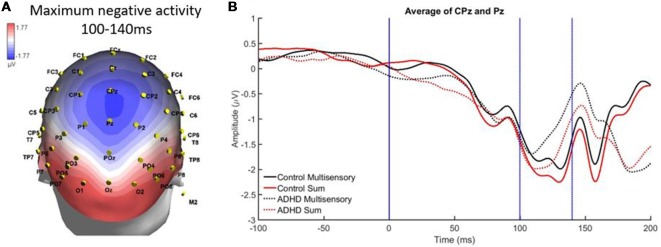
**(A)** Localized negative activity in response to audiovisual (AV) multisensory stimulus from 100 to 140 ms over CPz and Pz electrodes. **(B)** Graph highlighting negative activity from 100 to 140 ms over central-parietal brain regions, with an effect of condition (sum vs. multisensory) for both groups, as the sum waveform is significantly more negative than the multisensory waveform, indicating that multisensory integration (MSI) occurred in both controls and those with ADHD.

There was localized negative activity for the time period of 100–140 ms in the central parietal region (electrodes CPz and Pz). A significant effect of stimulus type, *F*_(1,19)_ = 16.293; *p* < 0.001; partial *η*^2^ = 0.462, indicated a significant difference between the average multisensory vs. sum waveforms in this latency window (see Figures 5A,B). This indicates that MSI occurred in both groups (ADHD and neurotypical controls) at this time point and region. There was not a main effect of group (*F* = 0.390) and there was no significant interaction (*F* = 0.9).

**Figure 6:**
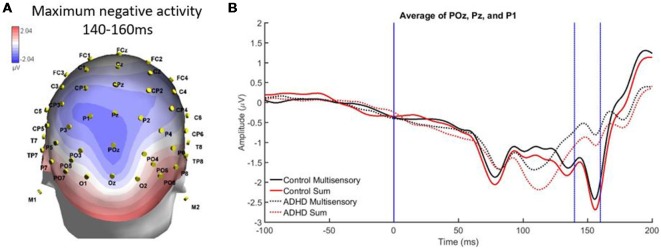
**(A)** Localized negative activity over Pz, P1, and POz from 140 to 60 ms in response to the AV multisensory stimulus. **(B)** Graph highlighting the negative activity from 140 to 160 ms over parietal-occipital brain regions. A main effect of group and stimulus type indicated that MSI occurred in both groups (controls and ADHD) at this latency and brain region, although event-related potential (ERP) activity was different between groups as controls had more negative activity.

A second region and time window of analysis was from 140 to 160 ms over parietal occipital regions (Pz, P1, and POz), which was observed as localized negative activity. This can be observed in the heat map in Figure 6A. Analysis revealed main effects of group, *F*_(1,19)_ = 7.295; *p* = 0.014; partial *η*^2^ = 0.277, and stimulus type, *F*_(1,19)_ = 5.420; *p* = 0.031; partial *η*^2^ = 0.222 (see Figures 6A,B). Specifically, the sum waveform was more negative than the multisensory waveform, and the controls had significantly more negative activity than the ADHD group. These findings indicate that while MSI occurred in both groups at this time and region, the ERP pattern was different in each group.

**Figure 7:**
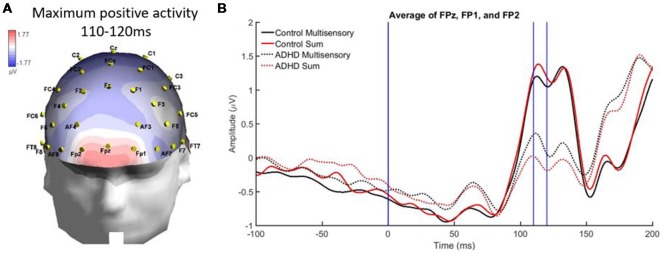
Panel **(A)** showing a localized positive activity from 110 to 120 ms over FPz, FP2, and FP1 in response to the AV multisensory stimulus. **(B)** Graph highlighting the positive activity from 110 to 120 ms over frontal regions with a condition by group interaction. The MSI waveform is more positive in those with ADHD and the opposite is seen in the control waveforms.

From 110 to 120 ms there was localized positive activity in pre-frontal regions (FPz, FP2, and FP1) as seen in Figure 7A. The analysis revealed no significant main effects and an interaction of stimulus type and group, *F*_(1,19)_ = 4.988; *p* = 0.038; partial *η*^2^ = 0.208. However, this interaction did not reveal MSI in either group. Instead, it showed that the sum waveform was more positive than the multisensory waveform for controls while the inverse was found for the ADHD group (i.e., equivalent differences between the sum and multisensory waveforms, but in different directions; see Figure 7B).

**Figure 8:**
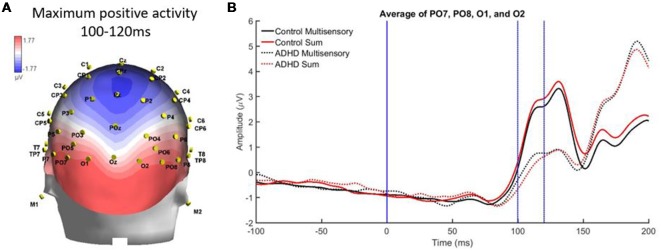
**(A)** Localized positive activity in electrodes PO7, O1, O2, and PO8 from 100 to 120 ms from the overall grand averaged heat maps. **(B)** Graph highlighting 100–120 ms over parietal occipital regions where an interaction between condition and group was approached.

Finally, there was a localized positive activity from 100 to 120 ms over parietal occipital regions (PO7, O1, O2, and PO8). This area is illustrated in Figure 8A. Analysis revealed no significant main effect, while a stimulus type by group interaction approached significance, *F*_(1,19)_ = 4.336; *p* = 0.051, *η*^2^ = 0.186, indicating a large effect size. This suggests a trend toward the pattern of electrical activity in response to the multisensory stimulus being different in both groups at this time point and brain region (see Figure 8B).

## Discussion

To our knowledge, this study was the first to examine multisensory processing in a population of young adults who have received a diagnosis of ADHD. Literature pertaining to ADHD is often assessing childhood ADHD, resulting in very little being known regarding adult ADHD and associated neurophysiological characteristics, particularly during AV multisensory processing. This is an important area of inquiry, as multisensory processing is fundamental to optimal human sensory functioning, and alterations to this neurological process may be related to some of the well-known behavioral characteristics of ADHD. This process is relevant to numerous environments that adults may interact within on a daily basis, such as sensory-rich environments while at work or school. By using a simple response time task while recording continuous EEG, distinct behavioral (i.e., response time) and neurophysiological (i.e., EEG) patterns of MSI were observed in both neurotypical controls and adults with ADHD.

### Behavioral Findings

In the response time task, both groups responded fastest to the multisensory stimulus, which was predicted due to the typical speeding of responses that occurs when individuals are presented with more than one percept. Previous studies utilizing a similar paradigm had found that an AV multisensory condition resulted in the shortest response time when compared to an auditory or visual unisensory condition (Brandwein et al., 2011, 2013, 2015). Interestingly, both ADHD and neurotypical control groups in the current study responded slowest to the unisensory auditory stimulus. Although unexpected due to the speed of typical auditory unisensory processing (Colavita, 1974), a similar finding occurred in previous studies utilizing similar conditions (A, V, and AV multisensory) in different special populations (Laurienti et al., 2004; Farid et al., 2018). Although the auditory condition had the longest response latencies in the current study, it had similar auditory response latencies to that of other studies hovering around 300 ms (Brandwein et al., 2011, 2013). This means that the visual and multisensory conditions resulted in faster responses than the auditory condition in our sample. The participants in this study had shorter response times to the visual and multisensory stimuli compared to prior results discussed in the literature, but similar auditory response latencies compared to previous research.

The type of auditory stimulus utilized for the present research may have influenced the behavioral results in this study, as an auditory verbalization was utilized as opposed to a pure-tone stimulus. When pure-tone auditory conditions are used, one would typically expect to see quicker responses than to those of a visual condition (Shelton and Kumar, 2010). Therefore, this indicates that the semantics involved in the auditory condition may have required longer processing times than needed in other auditory conditions utilizing a pure-tone stimulus, which explains the longer auditory response times. Other research has also elucidated that in certain multimodal paradigms, the visual stimulus dominates over the auditory stimulus and drives the multisensory interaction (Colavita, 1974). This possibly explains why the visual stimulus was the quickest unisensory condition in the paradigm utilized here. However, because this study was investigating MSI, where the auditory and visual stimuli should be semantically congruent for optimal integration, the verbalization of the word “red” was chosen as the auditory stimulus.

Differences in multisensory gain between groups was also assessed using Miller’s race model. This demonstrated that those with ADHD had greater violation of the race model in early time bin quantiles (i.e., where the multisensory score was smaller than the race model score), where neurotypical controls did not have violation in the early quantiles. This race model violation suggests that MSI likely contributed to the faster response times in the multisensory condition, as opposed to the faster of the two independently processed unisensory stimuli (Miller, 1982). Overall, the response time and race model data show similar findings with respect to differences in MSI between groups. That is, participants with ADHD had a trend towards responding faster and they violated the race model at an earlier quantile compared to the neurotypical participants. The above findings may be related to some of the symptomatology of ADHD such as impulsivity (Visser et al., 2014), as well as reflecting underlying neurological differences, allowing for those with ADHD to respond quicker to a given stimulus.

### Neurophysiological Findings

This study was the first of its kind to assess MSI in adults with ADHD and combined behavioral and neurophysiological measures. Based on methodology from previous literature assessing MSI, we found that specific patterns of MSI were apparent. MSI was found to have occurred in both study groups, however, there were some differences in how that activity occurred in each group (i.e., the patterns of MSI were not exactly the same). In particular, MSI occurred in both groups over central parietal regions from 100 to 140 ms. The parietal region is often discussed as being a sensory integration site (Brandwein et al., 2011), therefore, the results found here are similar to previous literature assessing MSI. Similarly, slightly left oriented multisensory processing was apparent over parietal-occipital electrodes; this is a similar left-lateralized pattern that has previously been identified in AV MSI (Molholm et al., 2002; Brandwein et al., 2013). From 140 to 60 ms, MSI occurred in both groups but a main effect of group indicated that ERP patterns over parieto-occipital regions differed in amplitude. This may be related to typical attentional alterations in those with ADHD, as MSI is influenced by the level of attentional allocation to individual stimulus components of a multisensory condition (Talsma et al., 2006). Interestingly, many of the ERP differences found in those with ADHD were in electrode groups pertaining to brain regions that previous literature has found to be structurally altered in those with ADHD (parietal, occipital, etc.; Valera et al., 2007; Proal et al., 2011; Duerden et al., 2012), suggesting that differences in brain region structure may result in differential neural processing of multisensory stimuli in associated electrodes.

Differences in brain activity between those with and without ADHD were also found in the present study. For instance, from 110 to 120 ms those with ADHD had significantly smaller ERPs than neurotypical controls. At this latency, controls also had a more positive sum waveform when compared to their multisensory waveform, while the opposite was true for the ADHD group, as their multisensory waveform was more positive than their sum waveform. A second time period of 100–120 ms demonstrated differences in brain activity over parietal-occipital regions, where the ADHD group again had much smaller ERPs than the neurotypical controls. The brain activity in response to the multisensory stimulus indicated that there is a difference in overall brain activity in this brain region and latency in those with ADHD. This may be related to the thinner cortical matter present in adults with ADHD, resulting in an attenuated or altered EEG signal when compared to neurotypical ERPs.

The electrodes pertaining to brain regions where a group difference was found in the amplitude of the ERPs in those with ADHD coincides with the regions that other research has shown to have altered brain structure in this population. For instance, the parietal and occipital regions of the brain are often thinner in those with ADHD (Durston et al., 2004; Valera et al., 2007; Proal et al., 2011; Duerden et al., 2012) and were also the regions where this study found significant differences in activity. Although analysis was limited to regions and specific time periods of maximal multisensory activity, there were also obvious differences in brain activity between the two groups in the amplitude and latencies of specific ERPs, which should be explored in future work. Additionally, we intentionally had participants maintain their usual medication regime, in order to reflect the state in which they function during their daily activities. Future work should compare individuals with ADHD in both medicated and non-medicated states.

A potential limitation of this study was the choice to test participants with ADHD while continuing their regular medication regime. This was done intentionally, in order to test adults with ADHD similarly to how they would function on a typical day, which includes taking medication pertaining to their ADHD. This allows for a clearer understanding of how these participants may react within environments that they are normally in, possibly including university or college lectures or common workplace settings. The fact that the response times of those on and off medication were so similar, suggests that medication did not have a significant influence on ADHD response times. With that in mind, future studies comparing adults with ADHD on and off medication may allow for further understanding of whether medication influences response time and multisensory processing.

## Conclusion

The main question this research investigated was whether those with ADHD processed AV multisensory stimuli similar to neurotypical individuals. Although the cortical activation differed between groups, there was evidence that those with ADHD did have MSI occur, with potentially associated shorter response latencies, which were described previously as a trend. This could indicate behavioral enhancement as a result of ADHD in adults, however the simple nature of the task did not require complex cognitive processing, only recognition, so the task does not allow conclusions to be made about the impact of these faster responses on cognitive function.

This is the first study to measure behavioral and neural characteristics of AV multisensory processing in young adults with ADHD. Extensions of this work may have application for the creation of technological supports, to promote optimal integration when in sensory-rich environments, as many work environments are multisensory. This is an important area of inquiry as a significant portion of the adult population has and will have ADHD. Future work should also consider utilizing a more complex task to assess multisensory processing capabilities in those with ADHD as well as conducting longitudinal and cross-sectional studies of the development of multisensory processing in individuals with ADHD.

## Author Contributions

All authors are listed and all have contributed significantly to the manuscript. HM, BM and PY made the conception and design of research and drafted the manuscript. HM performed experiments. HM, JB and AK analyzed data and prepared figures. HM, BM, CG, JB, AK, and PY interpreted results of experiments, edited and revised the manuscript.

## Conflict of Interest Statement

The authors declare that the research was conducted in the absence of any commercial or financial relationships that could be construed as a potential conflict of interest.
